# Segmental and tandem chromosome duplications led to divergent evolution of the *chalcone synthase* gene family in *Phalaenopsis* orchids

**DOI:** 10.1093/aob/mcy136

**Published:** 2018-08-02

**Authors:** Yi-Tzu Kuo, Ya-Ting Chao, Wan-Chieh Chen, Ming-Che Shih, Song-Bin Chang

**Affiliations:** 1 Department of Life Sciences, National Cheng Kung University, Tainan, Taiwan; 2 Agricultural Biotechnology Research Center, Academia Sinica, Taipei, Taiwan

**Keywords:** Chalcone synthase, gene family, gene duplication, expression profile, chromosome evolution, fluorescence *in situ* hybridization

## Abstract

**Background and Aims:**

Orchidaceae is a large plant family, and its extraordinary adaptations may have guaranteed its evolutionary success. Flavonoids are a group of secondary metabolites that mediate plant acclimation to challenge environments. Chalcone synthase (CHS) catalyses the initial step in the flavonoid biosynthetic pathway. This is the first chromosome-level investigation of the *CHS* gene family in *Phalaenopsis aphrodite* and was conducted to elucidate if divergence of this gene family is associated with chromosome evolution.

**Methods:**

Complete *CHS* genes were identified from our whole-genome sequencing data sets and their gene expression profiles were obtained from our transcriptomic data sets. Fluorescence *in situ* hybridization (FISH) was conducted to position five *CHS* genes to high-resolution pachytene chromosomes.

**Key Results:**

The five *Phalaenopsis CHS* genes can be classified into three groups, *PaCHS1*, *PaCHS2* and the tandemly arrayed three-gene cluster, which diverged earlier than those of the orchid genera and species. Additionally, pachytene chromosome-based FISH mapping showed that the three groups of *CHS* genes are localized on three distinct chromosomes. Moreover, an expression analysis of RNA sequencing revealed that the five *CHS* genes had highly differentiated expression patterns and its expression pattern-based clustering showed high correlations between sequence divergences and chromosomal localizations of the *CHS* gene family in *P. aphrodite*.

**Conclusions:**

Based on their phylogenetic relationships, expression clustering analysis and chromosomal distributions of the five paralogous *PaCHS* genes, we proposed that expansion of this gene family in *P. aphrodite* occurred through segmental duplications, followed by tandem duplications. These findings provide information for further studies of *CHS* functions and regulations, and shed light on the divergence of an important gene family in orchids.

## INTRODUCTION

Orchidaceae is a large plant family among the angiosperms, with >25 000 species that belong to >800 genera (Dressler, 1993). Orchids show high levels of variation in their floral morphologies, which make them, especially the *Phalaenopsis* cultivars, popular ornamentals in global horticultural markets. In addition, orchids are epiphytic, terrestrial or even lithophytic, and are renowned for their extraordinary acclimations. The evolutionary success of Orchidaceae may result from their diversified reproductive and ecological strategies (Givnish *et al.*, 2015).

Flavonoids are a group of plant secondary metabolites. They play numerous important roles in plants, such as flower pigmentation (anthocyanins), legume–rhizobial interactions (isoflavones), protection against UV radiation (flavonols), pathogen defence (isoflavonoids) and pollen fertility (Ferreyra *et al.*, 2012). Thus, they greatly enhance plant tolerance and adaptive ability. The flavonoid biosynthetic pathway includes the phenylpropanoid and flavonoid pathways.

Chalcone synthase (CHS) is a key enzyme in the phenylpropanoid biosynthetic pathway and is ubiquitous in plants. It functions in the initial step of the phenylpropanoid pathway and condenses three malonyl-CoA molecules with one 4-coumaroyl-CoA molecule to generate a naringenin chalcone, which is the precursor of various flavonoids. Owing to their importance in flavonoid production, *CHS* genes have been well studied in plant species such as *Petunia hybrida* (Koes *et al.*, 1989), *Zea mays* (Franken *et al.*, 1991; Han *et al.*, 2016), *Ipomoea purpura* (Durbin *et al*., 1995, 2000), *Gerbera hybrida* (Helariutta *et al.*, 1996; Deng *et al.*, 2014), *Arabidopsis thaliana* (Saslowsky *et al.*, 2000), *Dendranthema* species (Yang *et al.*, 2002), *Viola* species (van den Hof *et al.*, 2008), *Physcomitrella patens* (Koduri *et al.*, 2010), 12 species belonging to the Rosid clade (Zavala and Opazo, 2015) and *Oryza sativa* (Hu *et al.*, 2017).

To date, most of the examined plant gene families originated through gene duplication. Gene copies can arise via several mechanisms (Panchy *et al.*, 2016). Whole-genome duplication or the duplication of an entire chromosome results in the most dramatic form of gene duplication, but there are also small-scale sub-genomic duplication events, such as tandem duplication, segmental duplication, transposon-mediated duplication and retroduplication. The duplicated genes aid plant acclimation in the following three main ways: (1) they increase the production of beneficial products; (2) they may generate newly functional genes; and (3) they may specialize in spatial and developmental expression.

Gene duplication followed by divergence is a conspicuous feature of plant gene families, especially in genes related to plant secondary metabolism (Ober, 2005). For example, shifts in substrate specificity and catalytic reaction were observed in *GhCHS2* of *G. hybrida* (Helariutta *et al.*, 1996). Additionally, expression patterns of duplicate copies of *CHS* genes resulted in extensive differentiations in *I. purpura* (Durbin *et al.*, 2000), *Vitis vinifera* (Goto-Yamamoto *et al.*, 2002), *O. sativa* (Hu *et al.*, 2017), *Z. mays* (Han *et al.*, 2016) and *P. patens* (Koduri *et al.*, 2010). Those findings demonstrated that the duplicate *CHS* genes play specialized functional roles over the course of evolution. The structures of *CHS* genes examined to date have been conserved, and exhibited two exons and one intron, except for the *CHS* genes of *Antirrhinum majus* (Sommer and Saedler, 1986), which had two introns, and a few *CHS* genes of *Z. mays* and *P. patens*, which were intronless (Koduri *et al.*, 2010; Han *et al.*, 2016). Recent advances in high-throughput sequencing techniques and the construction of physical maps have made conducting genome-wide surveys easier. Thus, genome-wide identification, characterization, expression and distribution analyses of the *CHS* gene family have been conducted in *Z. mays* and *O. sativa* (Han *et al.*, 2016; Hu *et al.*, 2017), and the evolution of this gene family should be further investigated in other species.

Studies of the *CHS* gene family in orchids have been limited. Three *CHS* genes were cloned from the flower of the orchid *Bromheadia finlaysoniana* (Liew *et al.*, 1998). *OCHS3* was highly expressed in young leaves, which were flushed with anthocyanin, and was detected at much lower levels in faintly coloured flowers. Pitakdantham *et al.* (2010) also isolated a *CHS* gene from *Dendrobium* Sonia Earsakul, and it was highly expressed in young flowers. In *Phalaenopsis*, three *CHS* genes were isolated, and their expression patterns in floral tissues at different developmental stages were diverse (Han *et al.*, 2006). Of the three *CHS* genes, *PhCHS5* was most highly expressed and was the sole *CHS* gene responsible for pigment accumulation. Additionally, the orchid *CHS* genes were further grouped into two clades, Orchid CHS1 and Orchid CHS2 groups, based on amino acid sequences. Chomicki *et al.* (2015) demonstrated that two *CHS* genes under UV-B induction were expressed in orchid root tips for effective protection of the photosynthetic cortex. However, comprehensive investigations of the *CHS* genes in orchid species that analyse their gene duplications, distributions, evolutionary processes and functional diversifications in multiple tissues/organs at different developmental stages are scarce. In addition, numerous transcriptomic databases are available (Su *et al.*, 2013; Tsai *et al.*, 2013; Cai *et al.*, 2015; Chao *et al.*, 2017); we are currently conducting whole-genome sequencing, assembling and annotation to facilitate an overall survey of this gene family.

Here, a comprehensive study of the *CHS* gene family in *Phalaenopsis aphrodite* was conducted. The *CHS* genes were identified by searching transcriptomic data sets, and their phylogenetic relationships with *CHS* genes in other plants were revealed. Furthermore, the expression profiles in various tissues/organs and developmental stages, and chromosomal localizations were analysed. These results provide a foundation for further study of the *CHS* gene family in orchids and an example of gene localization and functional diversification in Orchidaceae.

## MATERIALS AND METHODS

### Identification of *CHS* genes in *Phalaenopsis* species and phylogenetic analysis

The *CHS* genes of *P. aphrodite* were identified from our orchid transcriptomics database, Orchidstra 2.0 (Chao *et al.*, 2017). The identities of amino acid and nucleotide sequences of the coding regions and sequence alignments were analysed using Vector NTI (Li and Moriyama, 2004). A phenetic tree was constructed based on the amino acid sequences of 60 CHSs from different plant taxa (Supplementary Data Table S1). The CHS sequences of *P. modesta* and *P. equestris* were downloaded from Orchidstra 2.0 (Chao *et al.*, 2017) and OrchidBase 2.0 (Tsai *et al.*, 2013), respectively, and the CHS sequences of the other plant species were downloaded from NCBI’s GenBank. These amino acid sequences were aligned using default the setting in ClustalW (Thompson *et al.*, 1994) implemented in MEGA7.0 (Kumar *et al.*, 2016). The *P. patens CHS* gene was used as the outgroup. Evolutionary history was inferred using the Neighbor–Joining (NJ) (Saitou and Nei, 1987) method. Evolutionary distances were computed using the Poisson correction method (Zuckerkandl and Pauling, 1965), and node support was assessed by bootstrap test (Felsenstein, 1985) with 1000 resampling replicates using MEGA7.0 (Kumar *et al.*, 2016).

### Cloning and labelling of DNA probes for fluorescence *in situ* hybridization (FISH) mapping

The cDNA sequences of *CHS* genes in *P. aphrodite* derived from the Orchidstra 2.0 database were first used as queries in a BLAST algorithm-based search of the assembled genomic shotgun sequences of *P. aphrodite*. Primers were designed using Primer3 to amplify DNA fragments that included or were close to the *CHS* coding regions from the genomic DNA of *P. aphrodite* (Supplementary Data Table S2). The CHS-containing DNA fragments were amplified by PCR, which was performed using 10 ng of genomic DNA, 1× PCR buffer, 0.4 mm of each dNTP, 0.3 μm each of forward and reverse primer, and 1.0 U of KOD FX Neo DNA polymerase (TOYOBO, Osaka, Japan) in a total reaction volume of 50 μL. The PCR step-down cycling conditions were as follows: 94 °C for 2 min; five cycles of 98 °C for 10 s and 70 °C for 3.5 min; five cycles of 98 °C for 10 s and 68 °C for 3.5 min; five cycles of 98 °C for 10 s and 65 °C for 3.5 min; 20 cycles of 98 °C for 10 s and 60 °C for 3.5 min; 68 °C for 7 min; and then 25° C for 10 s. The PCR products were then cloned into the pZeroback vector (TIANGEN Biotech, Beijing, China) and transformed into *Escherichia coli* DH5α.

Plasmid DNA was extracted using a Plasmid Miniprep Purification Kit (GMbiolab, Taichung, Taiwan), and 1.8 μg of plasmid DNA was labelled with either biotin-dUTP or digoxigenin-dUTP using standard nick translation following the manufacturer’s protocol (Roche, Basel, Switzerland). Three plasmid DNAs with a total DNA insert size of approx. 10 kb were mixed together and used as a probe for pachytene chromosome-based FISH mapping (Supplementary Data Table S2).

### Preparation of pachytene chromosomes and FISH mapping

Flower buds of *P. aphrodite* (2*n* = 2*x* = 38) with a size range of 8.50–9.20 mm were collected to obtain developing pollinia with chromosomes at the meiotic pachytene stage. Pachytene chromosomal spreads were prepared following the modified drop method, as previously described (Kuo *et al.*, 2016). Meiotic pachytene spreads with little to no cytoplasm and good pachytene chromosome spreading were selected and stored at 4 °C for later use.

The FISH was carried out as previously described, with some modifications (Zhong *et al.*, 1996). The selected slides were incubated at 37 °C overnight or at 65 °C for 30 min to air-dry the chromosomal spreads before FISH. The pre-hybridization treatment included 5 μg mL^–1^ pepsin for 20 min and freshly prepared formaldehyde buffer [1× phosphate-buffered saline (PBS), 50 mm MgCl_2_ and 1 % formaldehyde] for 10 min. Slides were then dehydrated through an ethanol series (70, 90 and 100 %). The hybridization mixture (10 % dextran sulphate sodium, 50 % formamide, 2× SSC, 0.25 % SDS and 100–200 ng of probe DNA) was boiled for 10 min, placed on ice for 5 min and added onto the slides. The slides were treated at 80 °C for 2.5 min on a hot plate and incubated in a humid chamber at 37 °C for 12–16 h. Digoxigenin-labelled probes were detected and amplified with sheep fluorescein isothiocyanate-conjugated anti-digoxigenin antibody (Roche) and anti-sheep–fluorescein isothiocyanate (VECTOR Laboratories, Burlingame, CA, USA), respectively, whereas biotin-labelled probes were detected and amplified with Avidin Texas Red (VECTOR Laboratories) and biotinylated anti-avidin D (VECTOR Laboratories), respectively. Finally, slides were dehydrated through an ethanol series (70, 90 and 100 %) and dried at 37 °C for 20 min. Chromosomes were counterstained with 1.5 μg mL^–1^ 4’,6-diamidino-2-phenylindole in mounting medium (VECTOR Laboratories). Images were captured using a Nikon DS Ri1 CCD camera attached to a Nikon ECLIPSE 80i microscope (Nikon, Tokyo, Japan). Images were adjusted using NIS-Elements D3.2 and Adobe Photoshop CS3. Chromosome straightening was carried out using ImageJ (https://imagej.nih.gov.ij/).

### Digital expression analysis

Orchidstra 2.0 (Chao *et al.*, 2017) is a publicly available transcriptomics resource for orchid species. Normalized transcripts per million (TPM) values derived from RNA sequencing (RNA-seq) were searched and downloaded from Orchidstra 2.0. Nine TPM values for each of the five *P. aphrodite CHS* genes examined in different tissues/organs or at different developmental stages were obtained. The downloaded TPM values were pre-processed into the logarithm of TPM to base 2 and converted into a heatmap by ClustVis (Metsalu and Vilo, 2015).

## RESULTS

### Identification of *CHS* genes in *Phalaenopsis* species

A great number of orchid sequences have become publicly available in databases, and they are valuable resources for orchid gene identification. In this study, we searched for *CHS* genes of *P. aphrodite* using an orchid transcriptomic database, Orchidstra 2.0 (Chao *et al.*, 2017). Five annotated *CHS* genes of *P. aphrodite* were identified and named *PaCHS1* to *PaCHS5* (Table 1). The length of their coding regions ranged from 388 (PaCHS2) to 395 (PaCHS1) amino acids, and these sequences were aligned (Supplementary Data Fig. S1). The defining amino acids of the CHS family and reported active site residues were all conserved in the five CHSs (Ferrer *et al.*, 1999). The identities of amino acid sequences ranged from 60 to 96 % (Table 1), and the identities of nucleotides in the coding regions were only slightly different from those of the amino acid sequences. The identities between the PaCHS1 sequence and the other four CHS sequences were relatively low, and ranged from 60 to 66 %. The highest protein sequence identity was between PaCHS4 and PaCHS5 (96 %). In addition, the sequence identities among PaCHS3, PaCHS4 and PaCHS5 were all higher than 87 %, and these sequences grouped into a cluster (Supplementary Data Fig. S2). Thus, based on the protein sequences, the five CHSs of *P. aphrodite* were classified into three groups, which consisted of PaCHS1, PaCHS2 and another three CHSs showing high sequence identities. In addition, the five *CHS* genes were all composed of two exons and one intron with similar gene structures and sizes.

**Table 1.** Sequence identities of the five *chalcone synthase* (*CHS*) genes in *P. aphrodite*

Sequence ID	Gene	Length of coding regions	Identity of amino acid and nucleotide sequences (%)				
PaCHS1	PaCHS2	PaCHS3	PaCHS4	PaCHS5
PATC124207	*PaCHS1*	395	–	60	63	66	64
PATC125513	*PaCHS2*	388	*64*	–	70	71	70
PATC159204	*PaCHS3*	391	*63*	*69*	–	92	91
PATC125905	*PaCHS4*	391	*64*	*69*	*88*	–	96
PATC124475	*PaCHS5*	393	*63*	*68*	*87*	*93*	–

The italic values are for nucleotide sequences.

### Chromosomal localizations of *CHS* genes in *P. aphrodite*

**Fig. 1.**
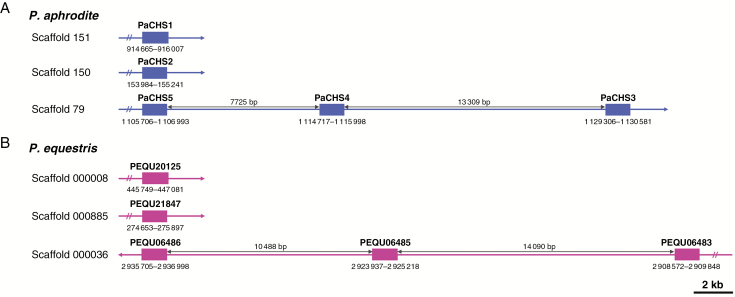
Physical positions of the *CHS* genes on the *Phalaenopsis* assembled genome sequences. The positions, scaffold numbers and intervals between the three tandemly arrayed *CHS* genes in (A) *P. aphrodite* and (B) *P. equestris* are indicated.

During evolution, tandem and segmental duplications are considered the main driving forces that expand gene families. Here, the distribution of the five *CHS* genes in chromosomes was investigated to determine the possible occurrence of duplication events that affected expansion of *CHS* genes in *P. aphrodite.* The coding sequences of the *CHS* genes were first mapped onto the assembled genome sequences of *P. aphrodite. PaCHS3*, *PaCHS4* and *PaCHS5* were tandemly arrayed on the same scaffold, scaffold 79 (Fig. 1A). *PaCHS1* and *PaCHS2* were located on scaffolds 151 and 150, respectively. However, neither genetic linkage nor physical maps have been established for *Phalaenopsis* species. Thus, FISH mapping was the technique chosen to investigate localizations of the *CHS* genes to *P. aphrodite* chromosomes.

**Fig. 2.**
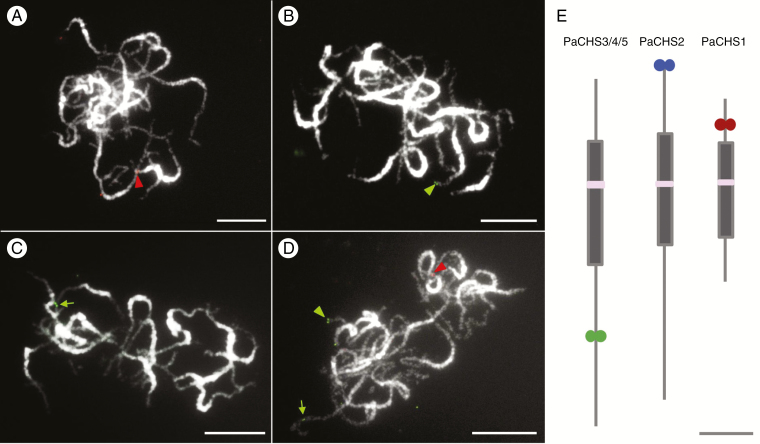
FISH mapping of *CHS* genes on pachytene chromosomes of *P. aphrodite*. *PaCHS1* (A, red arrowhead), *PaCHS2* (B, green arrowhead) and *PaCHS3/4/5* (C, green arrow) only showed distinct signals on a *P. aphrodite* pachytene chromosome. (D) A pool of the three CHS probes in (A–C). Chromosomes were stained with 4’,6-diamidino-2-phenylindole, and images were converted to black and white. Scale bar = 10 μm. (E) Ideogram of the three CHS-localized pachytene chromosomes. Grey and pink boxes indicate the heterochromatic region and centromere, respectively. The green and red circles represent the chromosomal localizations of the CHS probes. Scale bar in (E) represents 5 μm.

Meiotic pachytene chromosomes, instead of mitotic metaphase chromosomes, were applied to enhance FISH mapping resolution. DNA fragments including the *CHS* genes and nearby fragments were amplified, cloned and then used as scaffold-specific probes to detect their precise locations on pachytene chromosomes. The primers used to amplify the DNA fragments are listed in Supplementary Data Table S2. For all three probes, PaCHS1, PaCHS2 and PaCHS3/4/5, only one distinct signal was detected on one of the 19 *P. aphrodite* chromosomes (Fig. 2). Probe PaCHS1 was mapped to the euchromatic region of the short arm of a pachytene chromosome (Fig. 2A), whereas probe PaCHS2 was located at the end of the short arm of a chromosome (Fig. 2B). Probe PaCHS3/4/5, which represented three tandemly arrayed *CHS* genes, was detected within the euchromatic region of the long arm of a chromosome (Fig. 2C). Furthermore, a pool of the three probes showed that they were located on three different *P. aphrodite* chromosomes (Fig. 2D). The ideograms of the three *CHS* gene-containing chromosomes are illustrated in Fig. 2E.

### Distinct expression profiles of the *CHS* genes in *P. aphrodite*

**Fig. 3.**
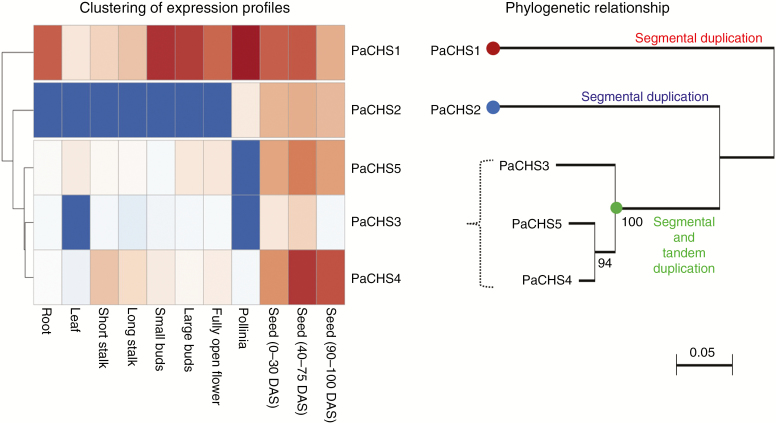
Clustering analysis based on expression profiles of the five *P. aphrodite CHS* genes and its association with sequence divergence. The heatmaps were generated from the normalized transcripts per kilobase million values derived from the RNA-seq analysis. Each column represents different tissues/organs at different developmental stages. DAS, days after sowing; long stalk, 1.5–3.0 cm; short stalk, <1.0 cm. We propose that the multiple copies of *PaCHS* genes, *PaCHS1*, *PaCHS*2 and *PaCHS3*, were generated by segmental duplication followed by the event whereby *PaCHS3/4/5* were arranged with additional tandem duplication.

Because the duplicated *CHS* genes showed divergent sequences and were located on different chromosomes, they may generate sub- or neo-functions. The expression patterns of genes within tissues or organs are usually correlated with their biological functions. The expression profiles of the five *P. aphrodite CHS* genes investigated by RNA sequencing (RNA-seq) were obtained from Orchidstra 2.0 (Chao *et al.*, 2017). The normalized expression data were converted to heatmaps for better visualization and their expression profiles in diverse tissues/organs at different developmental stages were also clustered (Fig. 3). *PaCHS1* showed constitutive elevated expression levels among organs and developmental stages. The highest and lowest *PaCHS1* expression levels were in pollinia and leaves, respectively, and its expression level decreased from small buds (1.3 cm) to fully open flowers, which resembled previously reported *PhCHS5* expression in a *Phalaenopsis* hybrid (Han *et al.*, 2006). *PaCHS2* had the lowest expression level of the five *CHS* genes, and it was only expressed in pollinia and seeds. Alternatively, the expression levels of *PaCHS3*, *PaCHS4* and *PaCHS5* were relatively lower than that of *PaCHS1* and were expressed mainly in seeds. *PaCHS3* and *PaCHS5* expression was not detected in pollinia. Moreover, the expression patterns of *PaCHS3*, *PaCHS4* and *PaCHS5* were similar and were distinct from those of *PaCHS1* and *PaCHS2*. Thus, the expression levels of the five *CHS* genes were highly differentiated and showed distinct expression patterns in *P. aphrodite*. Interestingly, we found that clustering based on the expression patterns of the five *CHS* genes was correlated with their sequence divergences and chromosomal distributions (Fig. 3).

### Phenetic analysis

**Fig. 4.**
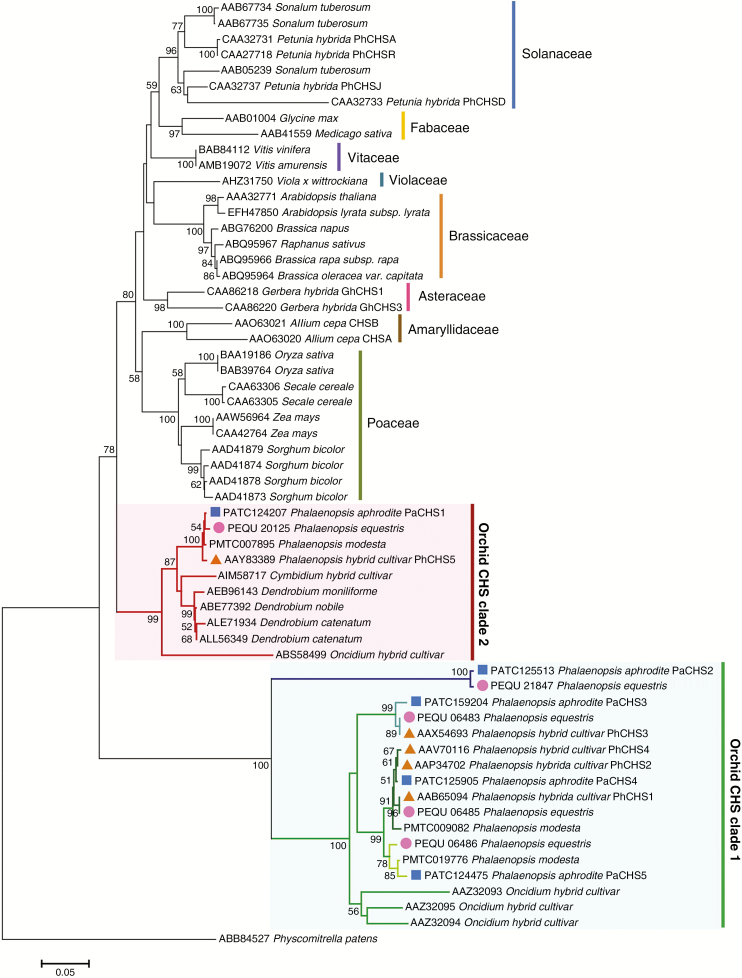
Neighbor–Joining tree depicting relationships among CHSs of orchids and other plant species. The Neighbor–Joining tree was constructed based on CHS amino acid sequences of *P. aphrodite* (blue squares), *P. equestris* (pink circles) and *P.* hybrid cultivar (orange triangles).

To verify the *CHS* gene lineage of *P. aphrodite* and other plant species, an NJ tree was constructed based on amino acid sequences. The detailed sequence information is listed in Supplementary Data Table S1. All orchid CHSs were classified into two major clades, Orchid clades 1 and 2 (Fig. 4), which was similar to previous findings (Chomicki *et al.*, 2015). Of the five *P. aphrodite* CHSs, only PaCHS1 was included in clade 2, and it was closely related to AAY83389 (PhCHS5 of a *Phalaenopsis* hybrid cultivar), which is associated with anthocyanin accumulation and photosynthetic root cortex protection in *Phalaenopsis* cultivars (Han *et al.*, 2006). In clade 1, PaCHS2 of *P. aphrodite* only clustered with PEQU_21847 of *P. equestris* and was distant from the other orchid CHSs in this clade. Its long branch length indicated a higher rate of sequence changes. Alternatively, the other three *P. aphrodite* CHSs, PaCHS3, PaCHS4 and PaCHS5, clustered in the *Phalaenopsis*-specific subclade. None of the orchid CHSs in the phenetic tree formed genus- or species-specific subclades, which indicates that the divergence of the orchid *CHS* genes might be earlier than that of the orchid genera or species. There are five homologous *CHS* genes in current studied *Phalaenopsis* species or cultivars (Fig. 4; Supplementary Data Fig. S3). In our analysis, we found five paralogous *CHS* genes and each had a corresponding orthologue between *P. aphrodite* and *P. equestris*, but that relationship was not found with the *Phalaenopsis* hybrid cultivar. There were two missing paralogues that corresponded to *PaCHS2* and *PaCHS5* in the *Phalaenopsis* hybrid cultivar, whereas three orthologues in that cultivar (*PhCHS1*, *PhCHS2* and *PhCHS4*) clustered with *P. aphrodite PaCHS4*. These results indicate that the cultivar has a mixed and complicated set of *CHS* genes donated by the parental species resulting from its hybrid origin.

## DISCUSSION

Flavonoids are important in plant development and acclimation. The *CHS* genes function in the initial step of the flavonoid biosynthetic pathway, and they are plant polyketide synthases (PKSs), which are involved in the biosynthesis of various secondary metabolites. This was the first integrated gene–chromosome–genome study of *CHS* genes in Orchidaceae, and five complete *CHS* genes were identified from the *P. aphrodite* transcriptomic data sets. In *P. aphrodite*, the *CHS* genes formed a relatively small gene family compared with other plant species; for example, there are three *CHS* genes in *Viola* species (van den Hof *et al.*, 2008), six in *Ipomoea* species (Durbin *et al.*, 2000), seven in *Sorghum bicolor* (Lo *et al.*, 2002), eight in both *Petunia hybrida* (Koes *et al.*, 1989) and *Pisum sativum* (Ito *et al.*, 1997), and up to 14 in *Z. mays* (Han *et al.*, 2016). However, only one complete *CHS* gene has been identified in *A. thaliana* (Saslowsky *et al.*, 2000).

Gene duplication is considered the mechanism that leads to expansion of gene families, and it is a prevailing feature in plant genomes. Generally, a high number of gene copies in a gene family is maintained through large-scale segmental duplication or small-scale tandem duplication during evolution. In *P. aphrodite*, *PaCHS3*, *PaCHS4* and *PaCHS5* formed a tandemly arrayed gene cluster, and the intervals between the three *CHS* genes were approx.13.3 kb (*PaCHS3* and *PaCHS4*) and 7.7 kb (*PaCHS4* and *PaCHS5*) (Fig. 1A). This arrangement was also observed in a closely related orchid, *P. equestris*, in which the three *CHS* genes were all positioned on scaffold 000036 (Fig. 1B). Furthermore, the three *P. aphrodite CHS* genes were clustered in a distinct subclade of clade 1 with the three CHSs of *P. equestris* (Fig. 4; Supplementary Data Fig. S3). Thus, the tandem array of three *CHS* genes was probably present in a common ancestor before speciation within *Phalaenopsis*. Tandem gene duplications represent a substantial proportion of all plant genes (Jander and Barth, 2007), including 17 % in *A. thaliana* (Arabidopsis Genome Initiative, 2000), 14 % in *O. sativa* (International Rice Genome Sequencing Project, 2005), 16 % in *Populustrichocarpa* (Tuskan *et al.*, 2006) and 35% in *Z. mays* (Messing *et al.*, 2004). In addition, most tandem duplications, including 75 and 79 % in *A. thaliana* and *O. sativa*, respectively, contain only two genes, and tandem gene arrays with more than three gene members are rare (Rizzon *et al.*, 2006). However, three tandemly arrayed *CHS* genes were found in our analysis, which indicated that tandem duplications most probably play a crucial role in expansion of *Phalaenopsis CHS* genes. The orchid genome harbours many remnants of one or more large-scale duplication events, but only 3.51 % of the *P. equestris* genome showed collinearity, which demonstrated a high degree of gene reshuffling after duplications (Cai *et al.*, 2015). Furthermore, FISH mapping showed that *PaCHS1*, *PaCHS2* and the gene cluster were located on three different chromosomes (Fig. 2). Thus, a large-scale genome duplication followed by reshuffling and sequence diversification might also have resulted in the duplications of *Phalaenopsis CHS* genes prior to the mentioned tandem duplication event. In *Z. mays*, no tandemly duplicated *CHS* genes have been found, and segmental duplication was the dominant contributor to expansion of the *Z. mays CHS* gene family (Han *et al.*, 2016). In contrast, no genes emerged through segmental duplications of the *O. sativa PKS* gene family, and expansion of the *O. sativa PKS* gene family was most probably caused by tandem duplications (Hu *et al.*, 2017). Consequently, the mechanisms underlying *CHS* gene duplications differed among plant species.

Chomicki *et al.* (2015) classified orchid *CHS* genes into two clades, Orchid CHS clade 1 and Orchid CHS clade 2, and these *CHS* genes identified from *Phalaenopsis* hybrid cultivars were included in our phylogenetic analysis. The NJ tree revealed that PaCHS1 clustered together with PhCHS5 (AAY83389), which belonged to Orchid CHS clade 2, whereas PhCHS1 (AAB65094), PhCHS2 (AAP34702), PhCHS3 (AAX54693) and PhCHS4 (AAV70116), members of Orchid CHS clade 2, clustered with the other four *P. aphrodite CHS* genes. Thus, based on the classification, PaCHS1 should belong to Orchid CHS clade 2 and the other four *P. aphrodite* CHSs should belong to Orchid CHS clade 2. However, the amino acid identities of PaCHS2 with the other four CHSs only ranged from 60 % (PaCHS1) to 70 % (PaCHS3, PaCHS4 and PaCHS5), and PaCHS2 only formed a subclade with a *P. equestris* CHS in clade 1 (Fig. 4; Supplementary Data Fig. S3). Additionally, *PaCHS2* was located on a distinct chromosome (Fig. 2), and was solely expressed in pollinia and seeds; no expression was detected in the other examined tissues or organs, which was a unique expression pattern. Therefore, based on the sequence identity, phylogenetic relationships, chromosomal localization and gene expression pattern, we strongly speculate that the *Phalaenopsis CHS* genes diverged into three groups instead of two.

Based on the phylogenetic relationships, *PaCHS1* was the only *CHS* gene in *P. aphrodite* allocated to Orchid CHS clade 2. PaCHS1 was closely related to PhCHS5 (AAY83389), which is associated with anthocyanin accumulation in the petals of *Phalaenopsis* cultivars (Han *et al.*, 2006). Additionally, the *PhCHS5* expression level decreased from small flower buds to fully open flowers, and this expression pattern was consistent with that of *PaCHS1* observed in RNA-seq data (Fig. 3). The highly similar expression patterns between *PaCHS1* and *PhCHS5* may indicate analogous gene functions. However, the number and expression patterns of *CHS* genes may not be the only factors that determine pigment accumulation and patterning in plants. No correlation has been found between flower colour and *CHS* genes in *Dendranthema* (Yang *et al.*, 2002), but gene inactivation and loss in the anthocyanin pathway of *Iochroma* (Solanaceae) is involved in flower pigmentation (Smith and Rausher, 2011; Smith *et al.*, 2013). Additionally, the combined expression of three MYB transcription factors that regulate pigmentation patterning was reported in *Phalaenopsis* cultivars (Hsu *et al.*, 2015). Therefore, even though *PaCHS1* is highly expressed in the stalks, flower buds and fully open flowers of *P. aphrodite*, the flowers are more or less white, with only a minor yellow pigmentation on the lips.

In addition to sequence divergences, spatial and developmental expression patterns of duplicate genes can differ. Distinct gene expression patterns may reflect different physical and chemical features, functions and regulations. The recently launched transcriptomic data sets provide valuable resources for analysing orchid functional genes in a genome scale. The normalized TPM values derived from *P. aphrodite* RNA-seq provided comparable data for analysing gene expression levels in various plant tissues or organs at different stages. The *P. aphrodite CHS* genes, with the exception of *PaCHS1*, which exhibited high levels of constitutive expression, showed relatively lower expression levels but had variable spatial distributions. This finding revealed functional diversification of duplicate *CHS* genes and confirmed that gene duplication was followed by divergence. In addition to the previously mentioned anthocyanin accumulation, CHSs also play crucial roles in plant resistance, which indicates that *CHS* gene expression results from stimulation of abiotic or biotic stresses, such as UV light (Han *et al.*, 2006), pathogens, low temperature and wounding (Dao *et al.*, 2011). Stress-induced *CHS* expression levels have been reported in *A. thaliana* (Feinbaum and Ausubel, 1988; Leyva *et al.*, 1995; Schenk *et al.*, 2000; Wade *et al.*, 2001), *Hordeum vulgare* (Christensen *et al.*, 1998), *A. majus* (Junghans *et al.*, 1993), *Daucus carota* (Glassgen *et al.*, 1998), *Brassica rapa* (Zhou *et al.*, 2007) and *S. bicolor* (Lue *et al.*, 1989). Most of the *Z. mays CHS* genes were up- or down-regulated after salicylic acid treatment, which is associated with abiotic stresses (Han *et al.*, 2016). In addition, the induction of *CHS* expression by the plant parasite, *Orobanche aegyptiaca*, which forms a physical connection with host roots, has been reported in diverse plant species (Griffitts *et al.*, 2004). Of the five *CHS* genes, *PaCHS1* is the most distant paralogue and is solely located on a single chromosome. The other four paralogous *CHS* genes were clustered in the *Phalaenopsis*-specific subclade of clade 1 (Fig. 4). In combination with chromosomal distributions, the four *CHS* genes were further classified into two groups, *PaCHS2* and the gene cluster composed of *PaCHS3*, *PaCHS4* and *PaCHS5*. The three genes were tandemly arrayed, which led us to speculate that expansion of the *CHS* gene family in *P. aphrodite* occurred through segmental duplications followed by tandem duplications (Fig. 3). In a previous study (Chomicki *et al.*, 2015), the five homologous *PhCHS* genes in a *Phalaenopsis* hybrid cultivar included, instead of five paralogues, a mixture of paralogues and orthologues due to sexual hybridization of wild species (Fig. 4; Supplementary Data Fig. S3). We suggest studying gene functions in a gene family using a wild species to avoid redundancy of orthologues and missing paralogues. In addition, *Cymbidium floriboundum* CHS in that study seems to be clustered in our *Phalaenopsis*-specific subclade. The amino acid sequences of *C. floriboundum* CHSs are partially available (<58 % of full-length of PaCHSs; 223 vs. 387–94 of amino acids); therefore, we did not include *C. floriboundum* CHSs in our analysis.

In this study, we classified the five *CHS* genes into three groups that were located on three distinct chromosomes. Based on the expression patterns of the five *CHS* genes, the clustering was correlated with their sequence divergences and chromosomal rearrangements. This correlation has not been found in other plant gene families, such as the *CHS* gene family of *Z. mays* (Han *et al.*, 2016) or the flavonol synthase gene family of *A. thaliana* (Owens *et al.*, 2008).

## SUPPLEMENTARY DATA

Supplementary data are available online at https://academic.oup.com/aob and consist of the following. Table S1: sequence information of CHSs used in the phylogenetic analysis. Table S2: sequences of primers used to amplify probe DNA for FISH mapping. Fig. S1: alignment of the amino acid sequences of the five CHSs identified in *P. aphrodite*. Fig. S2: chalcone synthase (CHS) phylogenetic relationships and their encoding gene structures in *P. aphrodite*. Fig. S3: phylogenetic relationships among the *Phalaenopsis* CHSs.

Supplementary Data Figure S1

Supplementary Data Figure S2

Supplementary Data Figure S3

Supplementary Data Table S1

Supplementary Data Table S2

Supplementary Data Captions
