# Supplementary figures and images for: Segmental and tandem chromosome duplications led to divergent evolution of the chalcone synthase gene family in Phalaenopsis orchids
Source: Ann Bot. 2018 Aug 2;123(1):69–77. doi: 10.1093/aob/mcy136 (PMC6344096; doi:10.1093/aob/mcy136)

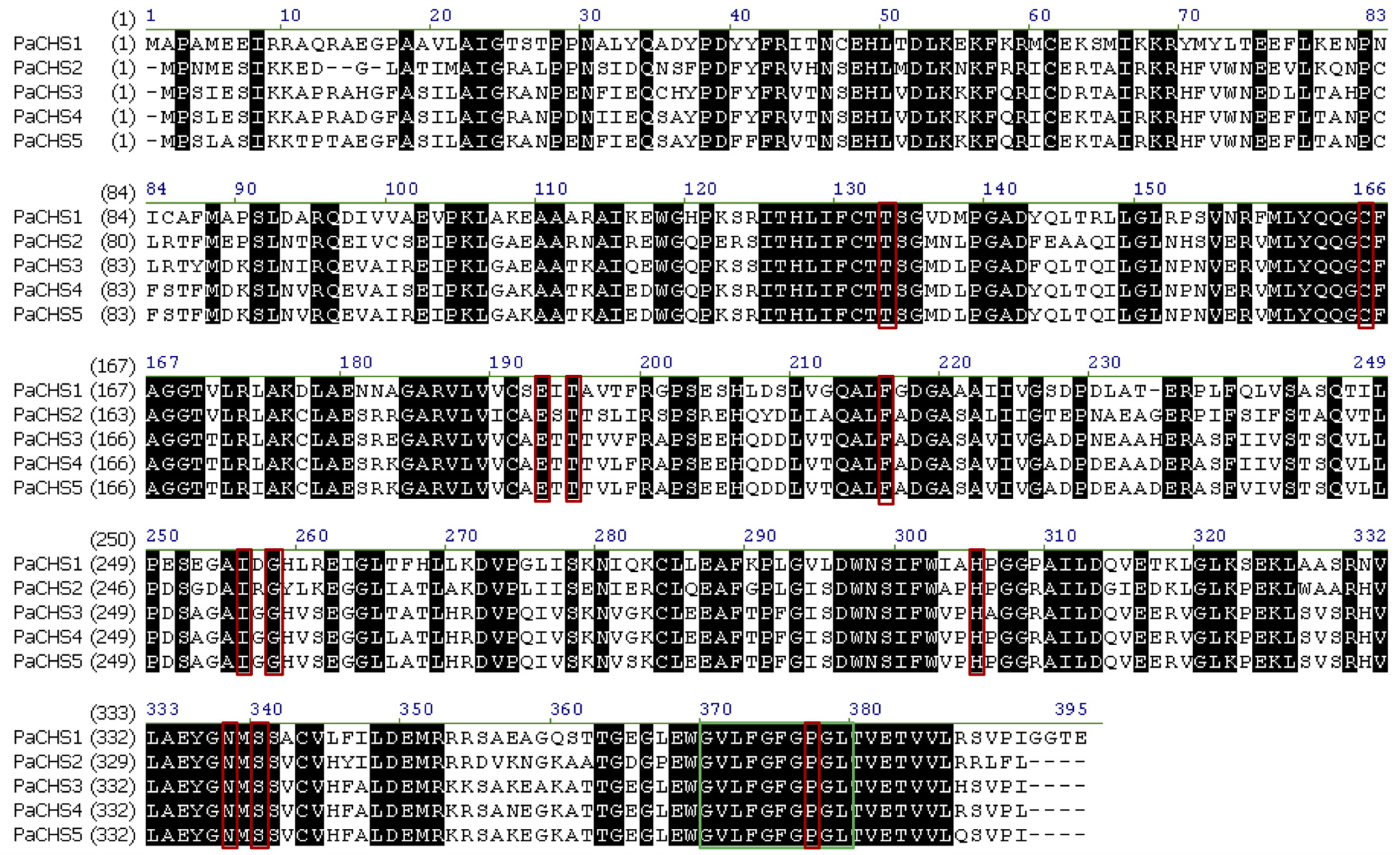

Supplement: Supplementary Data Figure S1 [file mcy136_suppl_aob-17790-s02.jpeg]

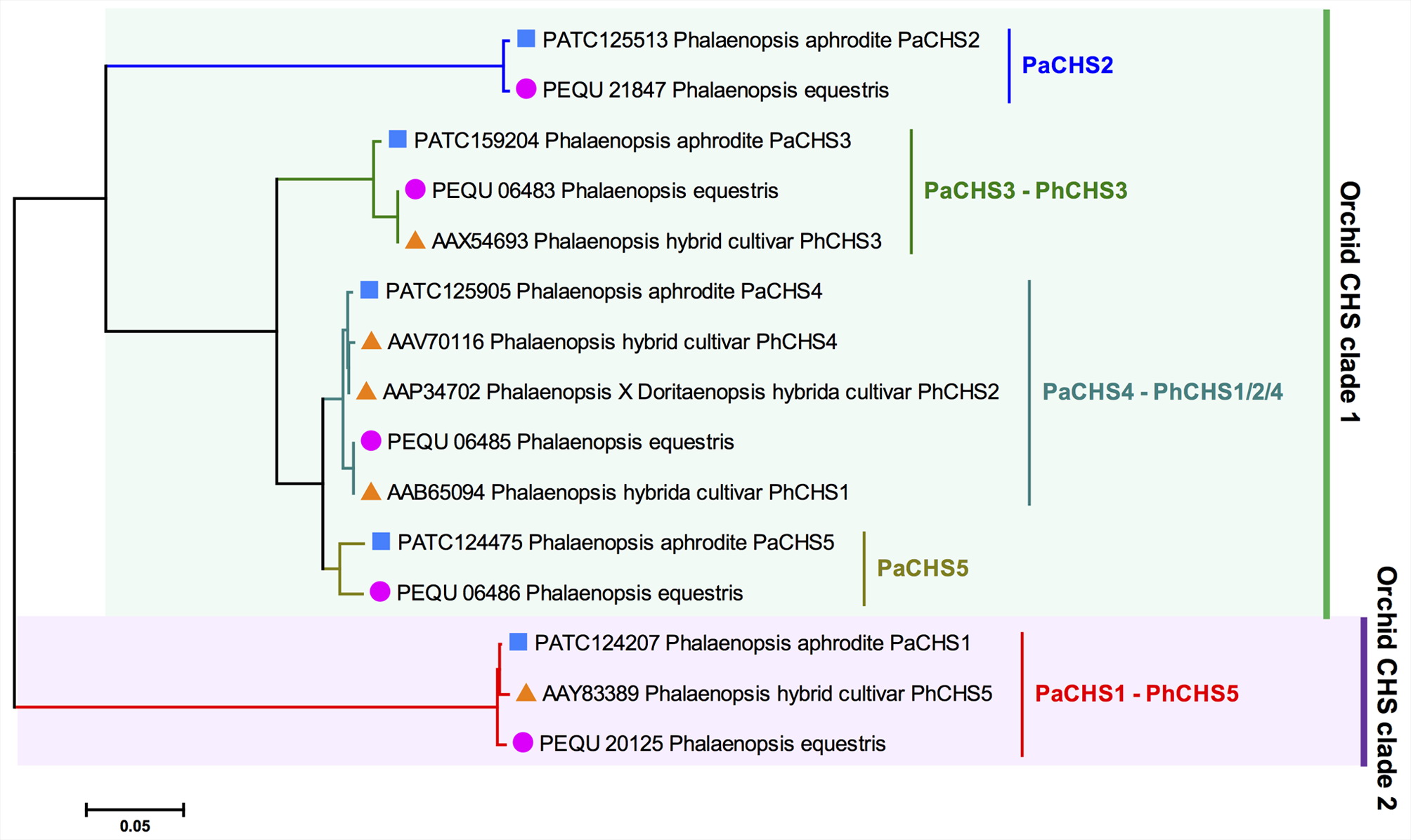

Supplement: Supplementary Data Figure S2 [file mcy136_suppl_aob-17790-s04.jpeg]

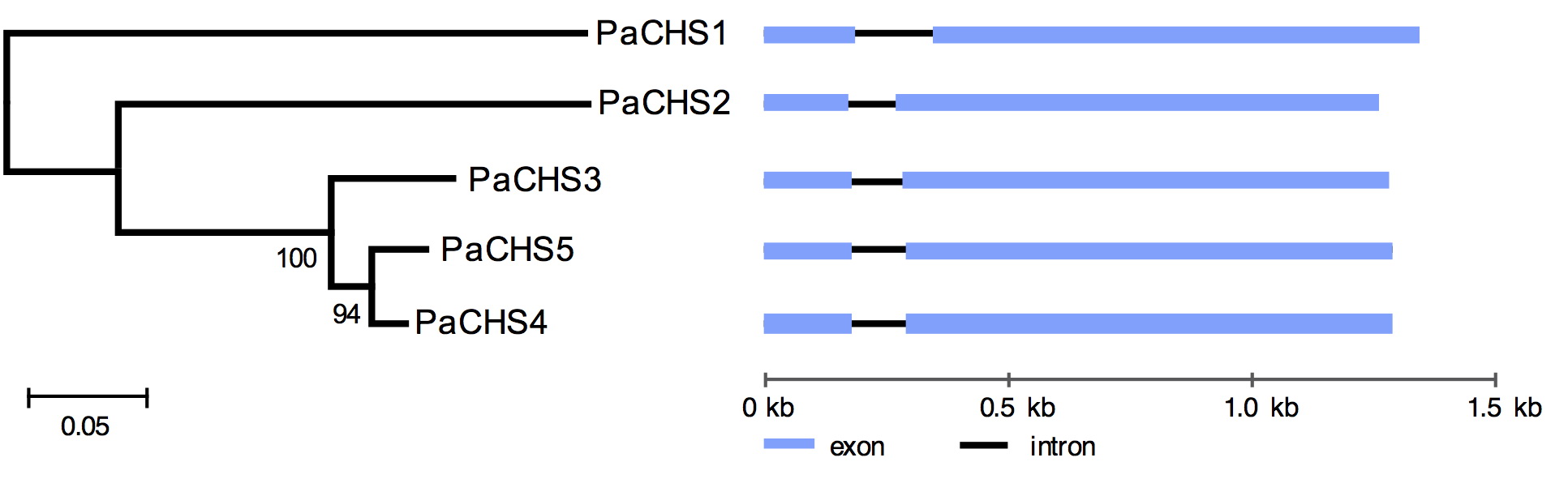

Supplement: Supplementary Data Figure S3 [file mcy136_suppl_aob-17790-s03.jpeg]
