# Supplementary material for: Segmental and tandem chromosome duplications led to divergent evolution of the chalcone synthase gene family in Phalaenopsis orchids
Source: Ann Bot. 2018 Aug 2;123(1):69–77. doi: 10.1093/aob/mcy136 (PMC6344096; doi:10.1093/aob/mcy136)
Supplement: Supplementary Data Table S1 [file mcy136_suppl_aob-17790-s06.docx]

Supplementary Table S2 Sequences of primers used to amplify probe DNA for FISH mapping

| Probe^a^ | DNA clone | Forward primer (5'-3') | Reverse primer (5'-3') | Amplicon size (kb) |
| --- | --- | --- | --- | --- |
| PaCHS1 | PaCHS1-1 | CCAGGTTTACGTGTTTGATATGG | TGCACCGTACTTTCCTGTGTT | 3.4 |
|  | PaCHS1-2 | TGTGTTCGAGGCAGGTGTAGT | CTTCTGCACACATCCCATTG | 3.6 |
|  | PaCHS1-3 | ACAAGTCGCGATACGAAAGC | CCATTGGTCCTCGCTAGGTAT | 2.8 |
| PaCHS2 | PaCHS2-1 | CTTGCGTTGTGGAGTTCAGTG | CCATCAGGTGCTCGCTGTTG | 3.8 |
|  | PaCHS2-2 | CATTGGGGTCAGTGGTCCAT | TTTACAGCTTTGGCGTGGTG | 2.1 |
|  | PaCHS2-3 | GTTCTTCCTGAGCAACGTCCTC | CGTTGGACCGATGAGAGATGATG | 3.5 |
| PaCHS3/4/5 | PaCHS3/4/5-1 | GGAGGAGACAAGGCAGGAGTT | ACATAGGCGGATTTGGGTTG | 4.0 |
|  | PaCHS3/4/5-2 | CGATATTAACAACTACTCCAACGGAAC | GAATTCAAAGTCACCTGCCCTA | 1.4 |
|  | PaCHS3/4/5-3 | GAAATTTGCCATCCCTCGAA | TTTTCGTCAGCATTTGTTTTATTTG | 4.4 |

^a^ Each probe was composed of three DNA clones with a sum DNA insert size of ~10 kb
