# Supplementary material for: Segmental and tandem chromosome duplications led to divergent evolution of the chalcone synthase gene family in Phalaenopsis orchids
Source: Ann Bot. 2018 Aug 2;123(1):69–77. doi: 10.1093/aob/mcy136 (PMC6344096; doi:10.1093/aob/mcy136)
Supplement: Supplementary Data Table S2 [file mcy136_suppl_aob-17790-s05.docx]

Supplementary Table S1 Sequence information of CHSs used in the phylogenetic analysis

| Family | Species | Accession no. | Database |
| --- | --- | --- | --- |
| Orchidaceae | *Phalaenopsis aphrodite* | PATC124207 | Orchidstra 2.0 |
|  |  | PATC125513 | Orchidstra 2.0 |
|  |  | PTTC159204 | Orchidstra 2.0 |
|  |  | PATC125905 | Orchidstra 2.0 |
|  |  | PATC124475 | Orchidstra 2.0 |
|  | *Phalaenopsis equestris* | PEQU_20125 | Orchidbase 3.0 |
|  |  | PEQU_06483 | Orchidbase 3.0 |
|  |  | PEQU_06485 | Orchidbase 3.0 |
|  |  | PEQU_06486 | Orchidbase 3.0 |
|  |  | PEQU_21847 | Orchidbase 3.0 |
|  | *Phalaenopsis modesta* | PMTC007895 | Orchidstra 2.0 |
|  |  | PMTC019776 | Orchidstra 2.0 |
|  |  | PMTC009082 | Orchidstra 2.0 |
|  | *Phalaenopsis* hybrid cultivar | AAX54693 | GenBank |
|  |  | AAV70116 | GenBank |
|  |  | AAY83389 | GenBank |
|  |  | AAB65094 | GenBank |
|  |  | AAP34702 | GenBank |
|  | *Dendrobium catenatum* | ALE71934 | GenBank |
|  |  | ALL56349 | GenBank |
|  | *Dendrobium moniliforme* | AEB96143 | GenBank |
|  | *Dendrobium nobile* | ABE77392 | GenBank |
|  | *Oncidium* hybrid cultivar | AAZ32093 | GenBank |
|  |  | AAZ32094 | GenBank |
|  |  | AAZ32095 | GenBank |
|  |  | ABS58499 | GenBank |
|  | *Cymbidium* hybrid cultivar | AIM58717 | GenBank |
| Poaceae | *Oryza sativa* | BAB39764 | GenBank |
|  |  | BAA19186 | GenBank |
|  | *Zea mays* | AAW56964 | GenBank |
|  |  | CAA42764 | GenBank |
|  | *Sorghum bicolor* | AAB05239 | GenBank |
|  |  | AAD41879 | GenBank |
|  |  | AAD41874 | GenBank |
|  |  | AAD41873 | GenBank |
|  | *Secale cereale* | CAA63306 | GenBank |
|  |  | CAA63305 | GenBank |
| Amaryllidaceae | *Allium cepa* | AAO63020 | GenBank |
|  |  | AAO63021 | GenBank |
| Solanaceae | *Solanum tuberosum* | AAB05239 | GenBank |
|  |  | AAB67734 | GenBank |
|  |  | AAB67735 | GenBank |
|  | *Petunia* *hybrida* | CAA32731 | GenBank |
|  |  | CAA32733 | GenBank |
|  |  | CAA32737 | GenBank |
|  |  | CAA27718 | GenBank |
|  |  | CAA32732 | GenBank |
| Asteraceae | *Gerbera hybrida* | CAA86218 | GenBank |
|  |  | CAA86220 | GenBank |
| Brassicaceae | *Arabidopsis thaliana* | AAA32771 | GenBank |
|  | *Arabidopsis lyrata* subsp*. lyrata* | EFH47850 | GenBank |
|  | *Brassica rapa* subsp. *rapa* | ABQ95966 | GenBank |
|  | *Brassica oleracea* var. *capitata* | ABQ95964 | GenBank |
|  | *Raphanus sativus* | ABQ95967 | GenBank |
| Vitaceae | *Vitis vinifera* | BAB84112 | GenBank |
|  | *Vitis amurensis* | AMB19072 | GenBank |
| Violaceae | *Viola x wittrochiana* | AHZ31750 | GenBank |
| Fabaceae | *Glycine max* | AAB01004 | GenBank |
|  | *Medicago sativa* | AAB41559 | GenBank |
| Funariaceae | *Physcomitrella patens* | ABB84527 | GenBank |
