# Supplementary material for: Segmental and tandem chromosome duplications led to divergent evolution of the chalcone synthase gene family in Phalaenopsis orchids
Source: Ann Bot. 2018 Aug 2;123(1):69–77. doi: 10.1093/aob/mcy136 (PMC6344096; doi:10.1093/aob/mcy136)
Supplement: Supplementary Data Captions [file mcy136_suppl_aob-17790-s01.docx]

**Supplementary Data captions**

**Table S1:** Sequence information of CHSs used in the phylogenetic analysis.

**Table S2:** Sequences of primers used to amplify probe DNA for FISH mapping.

**Fig. S1:** Alignment of the amino acid sequences of the five CHSs identified in *P. aphrodite*. The green and red boxes represent the defining amino acid of the gene family and conserved 12 residues of the active sites.

**Fig. S2:** Chalcone synthase (CHS) phylogenetic relationships and their encoding gene structures in *P. aphrodite*. The structure of all five *CHS* genes consisted of two exons (blue boxes) and one intron (black lines).

**Fig. S3:** Phylogenetic relationships among the *Phalaenopsis* CHSs. The neighbor-joining tree was constructed based on the amino acid sequences of the CHSs in *P. aphrodite* (*blue squares*), *P. equestris* (*pink circles*), and *P.* hybrid cultivar (*orange triangles*). The CHS orthologues between *P. aphrodite* and *P.* hybrid cultivar in each subclade are indicated.
